# Effect of genetic variants in cell adhesion pathways on the biochemical recurrence in prostate cancer patients with radical prostatectomy

**DOI:** 10.1002/cam4.2163

**Published:** 2019-04-16

**Authors:** Chia‐Cheng Yu, Lih‐Chyang Chen, Victor C. Lin, Chao‐Yuan Huang, Wei‐Chung Cheng, Ai‐Ru Hsieh, Ta‐Yuan Chang, Te‐Ling Lu, Cheng‐Hsueh Lee, Shu‐Pin Huang, Bo‐Ying Bao

**Affiliations:** ^1^ Division of Urology, Department of Surgery Kaohsiung Veterans General Hospital Kaohsiung Taiwan; ^2^ Department of Urology, School of Medicine National Yang‐Ming University Taipei Taiwan; ^3^ Department of Pharmacy College of Pharmacy and Health Care, Tajen University Pingtung Taiwan; ^4^ Department of Medicine Mackay Medical College New Taipei City Taiwan; ^5^ Department of Urology E‐Da Hospital Kaohsiung Taiwan; ^6^ School of Medicine for International Students I‐Shou University Kaohsiung Taiwan; ^7^ Department of Urology National Taiwan University Hospital, College of Medicine, National Taiwan University Taipei Taiwan; ^8^ Graduate Institute of Biomedical Sciences China Medical University Taichung Taiwan; ^9^ Research Center for Tumor Medical Science China Medical University Taichung Taiwan; ^10^ Drug Development Center China Medical University Taichung Taiwan; ^11^ Graduate Institute of Biostatistics China Medical University Taichung Taiwan; ^12^ Department of Occupational Safety and Health China Medical University Taichung Taiwan; ^13^ Department of Pharmacy China Medical University Taichung Taiwan; ^14^ Department of Urology Kaohsiung Medical University Hospital Kaohsiung Taiwan; ^15^ Graduate Institute of Medicine, College of Medicine Kaohsiung Medical University Kaohsiung Taiwan; ^16^ Department of Urology, Faculty of Medicine, College of Medicine Kaohsiung Medical University Kaohsiung Taiwan; ^17^ Institute of Biomedical Sciences National Sun Yat‐sen University Kaohsiung Taiwan; ^18^ Sex Hormone Research Center China Medical University Hospital Taichung Taiwan; ^19^ Department of Nursing Asia University Taichung Taiwan

**Keywords:** biomarker, CDH2, cell adhesion, prognosis, prostate cancer

## Abstract

The aberrant expression of cell adhesion molecules is a hallmark of epithelial‐to‐mesenchymal transition, resulting in the transformation of cancer cells to a more aggressive phenotype. This study investigated the association between genetic variants in cell adhesion pathways and the prognosis of patients with prostate cancer following radical prostatectomy (RP). A total of 18 haplotype‐tagging single‐nucleotide polymorphisms (SNPs) in eight cancer‐related adhesion molecules were genotyped in 458 prostate cancer patients, followed by the replication of the top SNPs in an additional set of 185 patients. Log‐rank test and multivariate Cox regression analysis adjusted for covariates were used to evaluate associations with the risk of biochemical recurrence (BCR) after RP. In the discovery set, four SNPs in *CDH2* were marginally associated with BCR. Among these, *CDH2* rs643555C > T was found to be associated with BCR in the replication set. Patients with rs643555TT genotype had a significantly shorter BCR‐free survival compared with those with CC/CT genotypes in the combined analysis (adjusted hazard ratio 1.78, 95% confidence interval 1.19‐2.67, *P* = 0.005). Additional analyses revealed that rs643555T was associated with higher expression of *CDH2*, and upregulated *CDH2* was correlated with tumor aggressiveness and shortened BCR‐free survival. In conclusion, rs643555C > T affects *CDH2* expression, and thus influences BCR in localized prostate cancer patients treated with RP. *CDH2* rs643555 may be a promising biomarker to identify patients at high risk of poor prostate cancer prognosis.

## 1 INTRODUCTION

Prostate cancer is the second most common cancer and the fourth leading cause of cancer death in men, worldwide.^1^ Most early stage prostate cancers tend to develop slowly and show an indolent clinical course. However, some prostate cancers display aggressive behavior and metastasize to other organs.^2^ While localized prostate cancer can be well controlled by active surveillance, radical prostatectomy (RP), or radiotherapy, metastatic tumors remain a lethal disease. Therefore, identification of key molecules and accurate prediction of patient prognosis are particularly important for prognostic and therapeutic purposes.

Recent evidence indicates that epithelial‐to‐mesenchymal transition (EMT) is a critical step for cancer progression. During EMT, polarized epithelial cells alter cell adhesion molecules and generate a new microenvironment, acquiring the aggressive behavior of metastatic competence, such as stem cell‐like features and treatment resistance.^3^ It is believed that the dysfunction of cell adhesion molecules, such as cadherins and integrins, is involved in cancer progression, based on the correlation of their expressions and tumor stage, metastasis, as well survival.^4^, ^5^

Given the important role of EMT in carcinogenesis, we hypothesized that genetic variants in cell adhesion molecules might influence the prognosis of prostate cancer. Therefore, we conducted a two‐stage study to investigate the impact of single‐nucleotide polymorphisms (SNPs) in eight cancer‐related cell adhesion molecules on the biochemical recurrence (BCR) in patients with localized prostate cancer after RP.

## 2 MATERIALS AND METHODS

### 2.1 Patient recruitment and data collection

A total of 643 patients, with histopathologically confirmed prostate cancer, that underwent RP were recruited from three Taiwan medical centers: Kaohsiung Medical University Hospital, Kaohsiung Veterans General Hospital, and National Taiwan University Hospital, as described previously.^6^ A two‐stage approach was applied to evaluate the effect of genetic variants in cell adhesion pathway genes on patient prognosis. Therefore, the study population was randomly divided into discovery and replication sets with a 7:3 ratio. The demographic data were collected through in‐person interviews using a structured questionnaire, which intends to identify individuals and/or families probably at‐risk for prostate cancer, and the clinicopathologic information was retrieved from patients’ medical records. All participants in the study were unrelated. BCR was defined according to two consecutive prostate‐specific antigen (PSA) measurements of 0.2 ng/mL or more after RP.^7^, ^8^ BCR‐free survival was defined as the duration from RP to the date of BCR. This study was approved by the institutional review board of Kaohsiung Medical University Hospital, and all participants signed the informed consent form according to institutional guidelines.

### 2.2 SNP selection and genotyping

Several adhesion molecules commonly participate in the cancer metastasis process, including CD276 molecule (CD276), CD6 molecule (CD6), CD8a molecule (CD8A), cadherin 2 (CDH2), claudin 11 (CLDN11), integrin subunit beta 1 (ITGB1), integrin subunit beta 7 (ITGB7), and poliovirus receptor (PVR). We initially selected 25 common tag SNPs in these cell adhesion genes using SNPinfo,^11^ based on the following criteria: a minor allele frequency of > 0.05 in the HapMap CHB (Han Chinese in Beijing) population, a pairwise linkage disequilibrium squared correlation coefficient (*r* ^2^) of > 0.8, whether they were potentially functional, and a maximum of five tag SNPs per gene. Genomic DNA was extracted from peripheral blood samples using the QIAamp DNA Blood Mini Kit (Qiagen, Valencia, CA, USA) according to manufacturer's instructions. Genotyping was carried out using Agena Bioscience iPLEX matrix‐assisted laser desorption/ionization time‐of‐flight mass‐spectrometry technology at the National Centre for Genome Medicine, Taiwan, as described previously.^12^ The average genotype call rate for these SNPs was 92.6% and the average concordance rate was 99.9% among 35 blind duplicated quality control samples. Any SNP that failed at assay design (*N* = 1), below a genotyping call rate of 80% (*N* = 4), or did not conform to Hardy‐Weinberg equilibrium (*P* < 0.005, *N* = 2), was removed. Thus, a total of 18 SNPs were included for further analyses.

### 2.3 Bioinformatics analysis

The regulatory annotation of the risk SNPs and their proxies (*r* ^2^ ≥ 0.8 in the Asian population from 1000 Genomes Project) was conducted by HaploReg v4.1.^13^ Expression quantitative trait loci (eQTL) analysis was performed by using Genotype‐Tissue Expression (GTEx).^14^ The prognostic significance of prostate cancer was analyzed using the publicly available GSE70769 microarray dataset.^15^

### 2.4 Statistical analysis

All statistical analyses were undertaken with Statistical Package for the Social Sciences software version 19.0.0 (IBM). Kaplan‐Meier analysis with log‐rank test was used to compare survival time between subgroups. Additive, dominant, and recessive genetic models were applied to analyze the prognostic effects of cell adhesion gene SNPs. The hazard ratios (HRs) and the corresponding 95% confidence intervals (CIs) were calculated by multivariate Cox regression analyses, which were adjusted for age, PSA at diagnosis, pathologic Gleason score, stage, and surgical margin. The combined HRs and 95% CIs were calculated by random effect models when heterogeneity of between‐set existed; otherwise a fixed effect model was used. All tests were two‐sided, and *P*‐values < 0.05 were regarded as statistically significant.

## 3 RESULTS

The clinicopathologic characteristics of patients in the discovery and replication sets are shown in Table 1. Most characteristics are comparable between the two sets. One hundred eighty‐four (40.2%) and 90 (48.6%) patients experienced BCR during the median follow‐up times of 54 and 74 months in the discovery and replication sets, respectively.

**Table 1:** Clinicopathologic characteristics of the study populations

Characteristics	Discovery	Replication
No. of patients	458	185
Median age, years (IQR)	66 (61‐70)	66 (61‐70)
Median PSA at diagnosis, ng/mL (IQR)	11.1 (7.1‐17.5)	11.0 (6.9‐18.7)
Gleason score, *N* (%)		
2	3 (0.7)	2 (1.1)
4	8 (1.8)	5 (2.8)
5	30 (6.6)	13 (7.2)
6	119 (26.3)	52 (28.7)
7	232 (51.2)	83 (45.9)
8	25 (5.5)	8 (4.4)
9	32 (7.1)	17 (9.4)
10	4 (0.9)	1 (0.6)
Pathologic stage, N (%)		
1	59 (13.1)	25 (13.9)
2	247 (54.9)	102 (56.7)
3	134 (29.8)	47 (26.1)
4	10 (2.2)	6 (3.3)
Surgical margin, *N* (%)		
Negative	241 (72.6)	104 (75.4)
Positive	91 (27.4)	34 (24.6)
Biochemical recurrence, *N* (%)	184 (40.2)	90 (48.6)
Median follow‐up, months	54	74

Abbreviations: IQR, interquartile range; PSA, prostate‐specific antigen.

**Figure 1:**
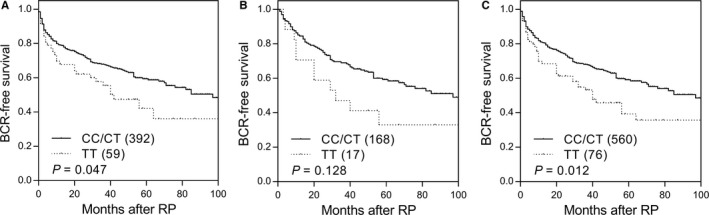
Kaplan‐Meier survival curves of biochemical recurrence‐free survival according to *CDH2* rs643555 genotypes in (A) discovery set, (B) replication set, and (C) combined analysis. Numbers in parentheses indicate the number of patients

Of the 18 SNPs in eight cell adhesion molecules analyzed in the discovery set, four SNPs in *CDH2* showed marginal association with BCR (Table S1). After adjusting for age, PSA at diagnosis, pathologic Gleason score, stage, and surgical margin, *CDH2* rs643555 remained significant (*P* = 0.039, Table 2 and Figure 1A). Consistent with the results of discovery set, *CDH2* rs643555 was found to be associated with BCR in the same direction in an independent replication set (*P* = 0.046, Table 2 and Figure 1B). In combined analysis, patients with rs643555 TT genotype had a significantly shorter BCR‐free survival compared with those with CC/CT genotypes (adjusted HR 1.78, 95% CI 1.19‐2.67, *P* = 0.005, Table 2 and Figure 1C).

**Table 2:** SNPs associated with BCR in patients receiving RP

Gene SNP	Discovery				Replication				Combined	
Genotype	*N*	BCR	HR (95% CI)	*P*	*N*	BCR	HR (95% CI)	*P*	HR (95% CI)	*P*
*CDH2* rs1944294										
AA	234	97	1.00		104	49	1.00		1.00	
AT	181	64	0.779 (0.524‐1.158)	0.217	67	31	0.808 (0.434‐1.506)	0.503	0.79 (0.56‐1.10)	0.16
TT	39	19	1.477 (0.818‐2.665)	0.196	14	10	**2.430 (1.087‐5.431)**	**0.031**	**1.76 (1.09‐2.83)**	**0.02**
AT/TT vs AA			0.885 (0.614‐1.274)	0.510			1.040 (0.597‐1.814)	0.889	0.93 (0.68‐1.26)	0.64
TT vs AA/AT			1.641 (0.929‐2.899)	0.088			**2.640 (1.221‐5.707)**	**0.014**	**1.94 (1.23‐3.07)**	**0.005**
*CDH2* rs3745045										
TT	132	57	1.00		60	31	1.00		1.00	
TC	242	87	0.952 (0.630‐1.437)	0.813	94	37	0.820 (0.424‐1.589)	0.557	0.91 (0.64‐1.30)	0.61
CC	82	38	0.955 (0.564‐1.617)	0.864	31	22	2.102 (0.996‐4.435)	0.051	1.24 (0.81‐1.91)	0.33
TC/CC vs TT			0.953 (0.645‐1.406)	0.807			1.103 (0.607‐2.002)	0.748	1.00 (0.72‐1.38)	0.98
CC vs TT/TC			0.985 (0.620‐1.565)	0.948			**2.351 (1.219‐4.534)**	**0.011**	1.31 (0.90‐1.92)	0.16
*CDH2* rs643555										
CC	207	85	1.00		85	41	1.00		1.00	
CT	185	64	0.793 (0.524‐1.202)	0.274	83	38	0.949 (0.522‐1.727)	0.865	0.84 (0.60‐1.18)	0.32
TT	59	29	1.490 (0.901‐2.462)	0.120	17	11	2.138 (0.943‐4.847)	0.069	**1.65 (1.07‐2.53)**	**0.02**
CT/TT vs CC			0.956 (0.662‐1.381)	0.812			1.126 (0.647‐1.960)	0.674	1.01 (0.74‐1.37)	0.97
TT vs CC/CT			**1.645 (1.024‐2.642)**	**0.039**			**2.191 (1.015‐4.731)**	**0.046**	**1.78 (1.19‐2.67)**	**0.005**
*CDH2* rs8084948										
TT	235	90	1.00		126	66	1.00		1.00	
TA	98	29	0.690 (0.400‐1.189)	0.182	51	20	0.489 (0.234‐1.022)	0.057	**0.61 (0.39‐0.95)**	**0.03**
AA	16	5	0.538 (0.185‐1.566)	0.255	8	4	0.939 (0.325‐2.718)	0.908	0.71 (0.34‐1.51)	0.38
TA/AA vs TT			0.660 (0.395‐1.103)	0.113			0.573 (0.302‐1.088)	0.089	**0.62 (0.42‐0.93)**	**0.02**
AA vs TT/TA			0.610 (0.212‐1.751)	0.358			1.123 (0.393‐3.206)	0.829	0.83 (0.39‐1.75)	0.62

Note

HRs were adjusted by age, PSA at diagnosis, pathologic Gleason score, stage, and surgical margin.

*P* < 0.05 are in boldface.

Abbreviations: BCR, biochemical recurrence; CI, confidence interval; HR, hazard ratio; RP, radical prostatectomy; SNP, single‐nucleotide polymorphism.

**Figure 2:**
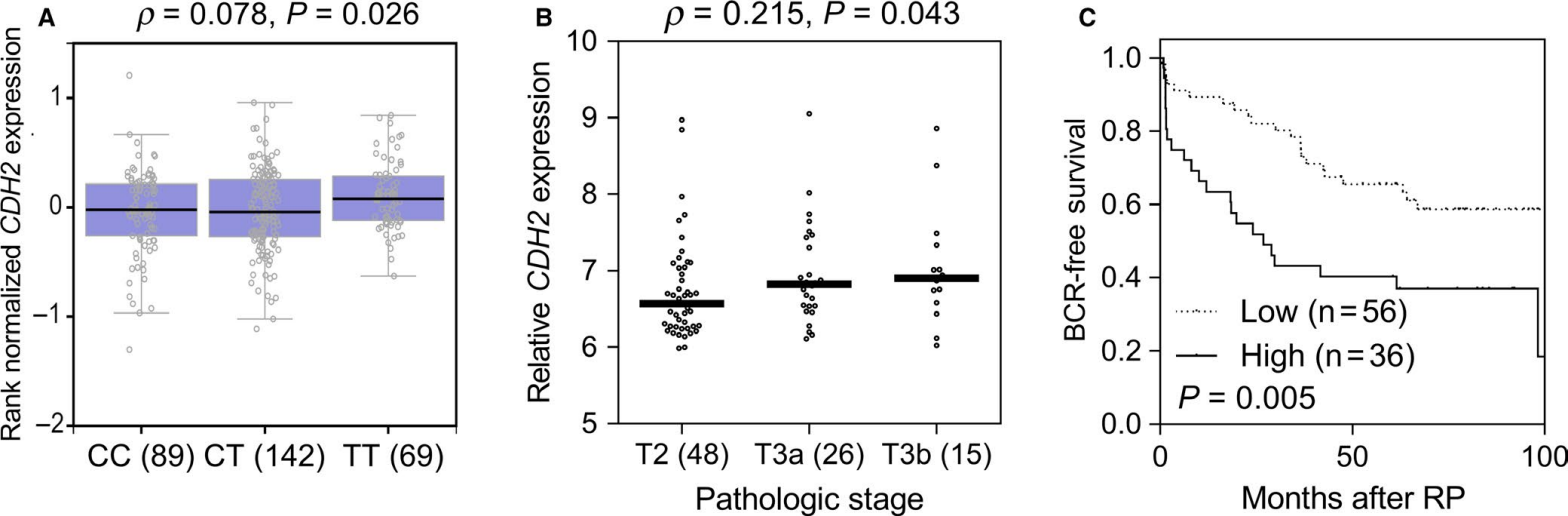
Functional analyses of *CDH2* rs643555. A, Association between rs643555 genotypes and *CDH2* expression in transformed human fibroblasts (GTEx dataset). B, *CDH2* is upregulated in advanced prostate cancers (GSE70769 dataset). C, Kaplan‐Meier survival curves of biochemical recurrence‐free survival according to *CDH2* expression levels. Patients were divided at the mean gene expression level into the low and high groups. Numbers in parentheses indicate the number of patients

According to the functional annotation using HaploReg v4.1, rs643555 is an eQTL for *CDH2*, and is predicted to alter multiple regulatory motifs (Table S2). In eQTL analysis from the GTEx dataset, the risk allele T of rs643555 showed increased *CDH2* expression in transformed human fibroblasts (*P* = 0.026, Figure 2A). We further investigated the prognostic effects of *CDH2* expression on BCR‐free survival after RP for prostate cancer. Based on the publicly available GSE70769 dataset, high expression of *CDH2* was associated with higher pathologic stages (*P* = 0.043, Figure 2B) and poorer BCR‐free survival (*P* = 0.005, Figure 2C).

## 4 DISCUSSION

In this exploratory study, we investigated associations between genetic variants in eight cancer‐related cell adhesion molecules and prostate cancer prognosis using a two‐stage study design. *CDH2* rs643555 was significantly associated with prostate cancer prognosis in both stages and remained significant after controlling for known risk factors. In addition, rs643555T risk allele was shown to upregulate the expression of *CDH2*, which was then linked to unfavorable BCR‐free survival outcomes, further supporting for the biological plausibility of our findings.

HaploReg identified that *CDH2* rs643555, an intronic variant, may be functional via a direct eQTL regulating the expression of *CDH2*.^16^ Several other potential causal variants in the proxy of rs643555 were also predicted to locate in enhancer histone marks and have regulatory effects on *CDH2* through eQTL. According to GTEx dataset, the risk allele T of rs643555 was correlated with increased expression of *CDH2*. CDH2, also known as N‐cadherin, is a transmembrane glycoprotein that mediates calcium‐dependent cell adhesion and is mainly expressed in multiple cell types, including nerve, vascular, myocardial, and mesenchymal cells.^17^ Evidence suggests that increased expression of CDH2 together with the loss of E‐cadherin (cadherin switching) plays an essential role during the progression of several human cancers,^18^ including prostate cancer.^19^ More importantly, aberrant CDH2 expression has been reported, not only in metastatic but also in castration‐resistant prostate cancer.^20^ Mechanistic studies demonstrated that CDH2 promotes prostate cancer cells EMT, stemness, and metastatic ability by activating the ErbB signaling pathway.^21^ Monoclonal antibodies against CDH2 inhibited androgen‐independent growth, local invasion, and metastasis in castration‐resistant prostate cancer models.^22^ Furthermore, a CDH2 antagonist, ADH‐1, is currently being used in clinical trials for treatment of CDH2‐expressing solid tumors.^23^ Together, this evidence further supports the hypothesis that CDH2 could be a promising therapeutic target for prostate cancer.

There are some inherent limitations in the present study. First, both study populations are Taiwanese; therefore, our findings may not be generalized to other ethnic groups. Second, we are unable to explore the biological mechanisms behind the SNPs of cell adhesion pathway genes and disease progression because the prostate cancer tissues from study participants were unavailable. Third, we used haplotype‐tagging SNPs to capture most of the genomic diversity, but the linked causal SNPs need to be further identified. Finally, the sample size of both cohorts is relatively small and does not have optimal power for discovering and replicating the associations. We had >80% power to detect a HR of 1.7 and 1.6 for BCR when assessing a SNP with minor allele frequency of 0.2 and 0.4, respectively, in the discovery set consisted of 458 patients. However, the association between *CDH2* rs643555 and prostate cancer progression was replicated across both sets of the study, which would reduce false‐positive findings. In addition, functional studies support the prognostic value of *CDH2* in prostate cancer. Further independent studies with larger number of patients from other ethnic groups and functional experiments are required to validate our findings.

In conclusion, by using a two‐stage study and bioinformatics analyses, we have identified that rs643555C > T acts as a risk factor of prostate cancer recurrence through increasing expression of *CDH2*. Our study provides new insights into the genetic variants in cell adhesion pathways underlying disease progression, and may offer a prognostic biomarker to the personalized management of prostate cancer.

## CONFLICT OF INTEREST

None declared.

## Data Availability

The data that support the findings of this study are available from the corresponding author upon reasonable request.

## References

[1] Bray F , Ferlay J , Soerjomataram I , Siegel RL , Torre LA , Jemal A . Global cancer statistics 2018: GLOBOCAN estimates of incidence and mortality worldwide for 36 cancers in 185 countries. CA Cancer J Clin. 2018;68(6):394‐424. 30207593 10.3322/caac.21492

[2] Cooperberg MR , Cowan J , Broering JM , Carroll PR . High‐risk prostate cancer in the United States, 1990–2007. World J Urol. 2008;26(3):211‐218. 18369637 10.1007/s00345-008-0250-7 PMC2948572

[3] Nieto MA , Huang RY , Jackson RA , Thiery JP . Emt: 2016. Cell. 2016;166(1):21‐45. 27368099 10.1016/j.cell.2016.06.028

[4] Cooper CR , Chay CH , Pienta KJ . The role of alpha(v)beta(3) in prostate cancer progression. Neoplasia. 2002;4(3):191‐194. 11988838 10.1038/sj.neo.7900224 PMC1531692

[5] Paul R , Ewing CM , Jarrard DF , Isaacs WB . The cadherin cell‐cell adhesion pathway in prostate cancer progression. Br J Urol. 1997;79(Suppl 1):37‐43. 9088271 10.1111/j.1464-410x.1997.tb00799.x

[6] Huang S‐P , Huang L‐C , Ting W‐C , et al. Prognostic significance of prostate cancer susceptibility variants on prostate‐specific antigen recurrence after radical prostatectomy. Cancer Epidemiol Biomarkers Prev. 2009;18(11):3068‐3074. 19900942 10.1158/1055-9965.EPI-09-0665

[7] Freedland SJ , Sutter ME , Dorey F , Aronson WJ . Defining the ideal cutpoint for determining PSA recurrence after radical prostatectomy. Urology. 2003;61(2):365‐369. 12597949 10.1016/s0090-4295(02)02268-9

[8] Huang C‐Y , Huang S‐P , Lin VC , et al. Genetic variants in the Hippo pathway predict biochemical recurrence after radical prostatectomy for localized prostate cancer. Sci Rep. 2015;5:8556. 25707771 10.1038/srep08556 PMC4338420

[9] Huang EY , Chang YJ , Huang SP , et al. A common regulatory variant in SLC35B4 influences the recurrence and survival of prostate cancer. J Cell Mol Med. 2018;22(7):3661‐3670. 29682886 10.1111/jcmm.13649 PMC6010704

[10] Huang S‐P , Lévesque E , Guillemette C , et al. Genetic variants in microRNAs and microRNA target sites predict biochemical recurrence after radical prostatectomy in localized prostate cancer. Int J Cancer. 2014;135(11):2661‐2667. 24740842 10.1002/ijc.28904

[11] Xu Z , Taylor JA . SNPinfo: integrating GWAS and candidate gene information into functional SNP selection for genetic association studies. Nucleic Acids Res. 2009;37(Web Server issue):W600‐W605. 19417063 10.1093/nar/gkp290 PMC2703930

[12] Huang C‐N , Huang S‐P , Pao J‐B , et al. Genetic polymorphisms in oestrogen receptor‐binding sites affect clinical outcomes in patients with prostate cancer receiving androgen‐deprivation therapy. J Intern Med. 2012;271(5):499‐509. 21880074 10.1111/j.1365-2796.2011.02449.x

[13] Ward LD , Kellis M . HaploReg v4: systematic mining of putative causal variants, cell types, regulators and target genes for human complex traits and disease. Nucleic Acids Res. 2016;44(D1):D877‐D881. 26657631 10.1093/nar/gkv1340 PMC4702929

[14] Consortium GT . The Genotype‐Tissue Expression (GTEx) project. Nat Genet. 2013;45(6):580‐585. 23715323 10.1038/ng.2653 PMC4010069

[15] Ross‐Adams H , Lamb AD , Dunning MJ , et al. Integration of copy number and transcriptomics provides risk stratification in prostate cancer: A discovery and validation cohort study. EBioMedicine. 2015;2(9):1133‐1144. 26501111 10.1016/j.ebiom.2015.07.017 PMC4588396

[16] Westra H‐J , Peters MJ , Esko T , et al. Systematic identification of trans eQTLs as putative drivers of known disease associations. Nat Genet. 2013;45(10):1238‐1243. 24013639 10.1038/ng.2756 PMC3991562

[17] Hatta K , Takeichi M . Expression of N‐cadherin adhesion molecules associated with early morphogenetic events in chick development. Nature. 1986;320(6061):447‐449. 3515198 10.1038/320447a0

[18] Maeda M , Johnson KR , Wheelock MJ . Cadherin switching: essential for behavioral but not morphological changes during an epithelium‐to‐mesenchyme transition. J Cell Sci. 2005;118(Pt 5):873‐887. 15713751 10.1242/jcs.01634

[19] Jaggi M , Nazemi T , Abrahams NA , et al. N‐cadherin switching occurs in high Gleason grade prostate cancer. Prostate. 2006;66(2):193‐199. 16173043 10.1002/pros.20334

[20] Jennbacken K , Tesan T , Wang W , Gustavsson H , Damber JE , Welen K . N‐cadherin increases after androgen deprivation and is associated with metastasis in prostate cancer. Endocr Relat Cancer. 2010;17(2):469‐479. 20233707 10.1677/ERC-10-0015

[21] Wang M , Ren D , Guo W , et al. N‐cadherin promotes epithelial‐mesenchymal transition and cancer stem cell‐like traits via ErbB signaling in prostate cancer cells. Int J Oncol. 2016;48(2):595‐606. 26647992 10.3892/ijo.2015.3270

[22] Tanaka H , Kono E , Tran CP , et al. Monoclonal antibody targeting of N‐cadherin inhibits prostate cancer growth, metastasis and castration resistance. Nat Med. 2010;16(12):1414‐1420. 21057494 10.1038/nm.2236 PMC3088104

[23] Perotti A , Sessa C , Mancuso A , et al. Clinical and pharmacological phase I evaluation of Exherin (ADH‐1), a selective anti‐N‐cadherin peptide in patients with N‐cadherin‐expressing solid tumours. Ann Oncol. 2009;20(4):741‐745. 19190075 10.1093/annonc/mdn695

