# Supplementary material for: Effect of genetic variants in cell adhesion pathways on the biochemical recurrence in prostate cancer patients with radical prostatectomy
Source: Cancer Med. 2019 Apr 16;8(6):2777–83. doi: 10.1002/cam4.2163 (PMC6558504; doi:10.1002/cam4.2163)
Supplement: Supplementary file 1 [file CAM4-8-2777-s001.doc]

**Table S1. Genotyped SNPs and the *P* values of their association with biochemical recurrence after radical prostatectomy**

| Gene | SNP ID | Chromosome | Position | Allele | MAF | HWE | BCR | | |
| --- | --- | --- | --- | --- | --- | --- | --- | --- | --- |
|  |  |  |  |  |  |  | Additive | Dominant | Recessive |
| *CD276* | rs11552770 | 15 | 71789046 | T>C | 0.140 | 0.835 | 0.265 | 0.201 | - |
| *CD276* | rs74026254 | 15 | 71768905 | C>G | 0.117 | 0.906 | 0.265 | 0.231 | - |
| *CD6* | rs3019548 | 11 | 60505515 | T>C | 0.082 | 0.737 | 0.981 | 0.923 | - |
| *CD6* | rs4939483 | 11 | 60526293 | A>T | 0.272 | 0.372 | 0.415 | 0.272 | 0.871 |
| *CD6* | rs2074230 | 11 | 60540380 | C>T | 0.100 | 0.613 | 0.555 | 0.344 | - |
| *CD8A* | rs13023213 | 2 | 86875454 | T>C | 0.074 | 0.523 | 0.336 | 0.367 | - |
| *CD8A* | rs1051386 | 2 | 86865584 | A>G | 0.182 | 0.382 | 0.626 | 0.438 | - |
| *CDH2* | rs1944294 | 18 | 23870449 | A>T | 0.281 | 0.645 | 0.714 | 0.703 | **0.108** |
| *CDH2* | rs1220020 | 18 | 23940618 | G>T | 0.335 | 0.740 | 0.768 | 0.764 | 0.247 |
| *CDH2* | rs3745045 | 18 | 23797660 | T>C | 0.442 | 0.103 | 0.740 | 0.447 | **0.130** |
| *CDH2* | rs643555 | 18 | 23857664 | C>T | 0.332 | 0.111 | 0.534 | 0.665 | **0.047** |
| *CDH2* | rs8084948 | 18 | 23970784 | T>A | 0.184 | 0.233 | 0.170 | **0.110** | - |
| *CLDN11* | rs6794467 | 3 | 171630685 | C>A | 0.375 | 0.836 | 0.720 | 0.978 | 0.490 |
| *ITGB1* | rs7358223 | 10 | 33269789 | A>G | 0.173 | 1.000 | 0.848 | 0.453 | - |
| *ITGB1* | rs2488320 | 10 | 33238917 | T>C | 0.239 | 0.443 | 0.503 | 0.854 | - |
| *ITGB7* | rs11170466 | 12 | 51872126 | C>T | 0.140 | 0.592 | 0.366 | 0.732 | - |
| *PVR* | rs7250339 | 19 | 49837452 | A>G | 0.438 | 1.000 | 0.608 | 0.940 | 0.409 |
| *PVR* | rs1058402 | 19 | 49842454 | G>A | 0.083 | 0.390 | 0.348 | 0.417 | - |

Abbreviations: SNP, single nucleotide polymorphism; BCR, biochemical recurrence; MAF, minor alleles frequency; HWE, Hardy-Weinberg equilibrium.

*P* values for log-rank test.

*P* < 0.15 is in boldface.

**Table S2. Regulatory annotation of variants linked with *CDH2* rs643555**

| Chromosome | Position | LD (r²) | SNP ID | Reference allele | Alternate allele | ASN frequency | Variant type | Promoter histone marks | Enhancer histone marks | DNAse | Proteins bound | eQTL hits | Motifs changed |
| --- | --- | --- | --- | --- | --- | --- | --- | --- | --- | --- | --- | --- | --- |
| 18 | 27999349 | 0.87 | rs665781 | C | T | 0.37 | intronic |  |  |  |  |  | Cdc5,ERalpha-a,RXRA |
| 18 | 28001809 | 0.89 | rs584936 | G | A | 0.37 | intronic |  |  |  |  |  | Foxa,Foxc1,Foxd1, Foxf1,Foxj1,Foxk1, Foxo,Foxq1,HDAC2, Sox,TCF12,TEF |
| 18 | 28002325 | 0.87 | rs597591 | A | C | 0.37 | intronic |  | BRN, VAS |  | GATA2 | 1 hit | Pax-4, Pou3f1,Pou3f2, SETDB1 |
| 18 | 28003928 | 0.9 | rs614966 | G | A | 0.37 | intronic | STRM | FAT, STRM, MUS, BRN, LNG, VAS, BONE | VAS |  | 1 hit | CEBPG,SRF |
| 18 | 28005299 | 0.86 | rs1122356 | A | G | 0.36 | intronic |  | FAT, MUS, BRN, HRT, LNG, VAS, BONE | ESDR,IPSC,HRT | CTCF, RAD21 | 1 hit | SMC3,TCF12 |
| 18 | 28009427 | 0.96 | rs623234 | C | T | 0.37 | intronic |  | ESDR |  |  |  | Foxp1,Sox |
| 18 | 28010971 | 0.93 | rs1220146 | C | T | 0.37 | intronic |  | ESDR |  |  |  | Irf,Maf,PLZF |
| 18 | 28016930 | 0.96 | rs490820 | C | T | 0.36 | intronic |  |  |  |  |  | COMP1,Foxd3,Nanog, Pbx-1, Pou2f2,Pou3f2, Pou5f1,Sox,TATA |
| 18 | 28019738 | 0.96 | rs673008 | G | A | 0.36 | intronic |  | BRST, MUS, SKIN, LIV, LNG, BONE | BRST,SKIN,GI,PANC, BRST,MUS |  | 1 hit | DMRT3,Hoxb6,Sox |
| 18 | 28023121 | 0.96 | rs656642 | C | G | 0.36 | intronic |  |  |  |  | 1 hit | DMRT4 |
| 18 | 28023702 | 1 | **rs643555** | C | T | 0.37 | intronic |  |  |  |  | 1 hit | Cphx,Foxa,Foxj2,Gfi1,Mef2 |
| 18 | 28023755 | 0.99 | rs576467 | C | T | 0.37 | intronic |  |  |  |  |  | Foxj2,Hoxa10,Hoxa9, Hoxb6,Ncx,Pdx1, Pou3f2,Sox,p300 |
| 18 | 28032498 | 0.95 | rs568575 | G | C | 0.36 | intronic |  | ESDR, MUS, BRN, HRT, LNG, VAS, BONE |  |  | 1 hit | Foxa,Foxi1,Foxj2, Foxk1,Foxl1,Foxo, HDAC2,Mef2,Nanog, Pou1f1,Pou2f2,Pou5f1,TATA,TCF12,TEF, p300 |
| 18 | 28033475 | 0.99 | rs539075 | T | G | 0.37 | intronic |  | ESDR, BRN, HRT | ESDR |  | 1 hit | Foxp1,Nkx3 |
| 18 | 28034070 | 0.95 | rs533602 | T | C | 0.36 | intronic |  | ESDR, IPSC, ESC, BRN, HRT |  |  | 1 hit | Ets,Nrf1,Sox,TATA, TR4 |
| 18 | 28037661 | 0.94 | rs8087457 | A | C | 0.37 | intronic |  | ESC, ESDR, IPSC, BRN, HRT | ESDR, OVRY |  |  | Isl2,Pax-6 |
| 18 | 28038303 | 0.96 | rs1238755 | T | G | 0.37 | intronic |  | ESDR, BRN, HRT |  |  |  | E2A,Ik-2,Ik-3,Mxi1,Myf,Pitx2, ZEB1 |
| 18 | 28044102 | 0.8 | rs1234682 | A | G | 0.38 | intronic |  | ESDR, ESC, BRN |  |  |  | GATA |
